# The spread of *Colletes hederae* Schmidt & Westrich, 1993 continues – first records of this plasterer bee species from Slovakia and the Czech Republic

**DOI:** 10.3897/BDJ.9.e66112

**Published:** 2021-04-23

**Authors:** Petr Bogusch, Jozef Lukáš, Martin Šlachta, Jakub Straka, Peter Šima, Jan Erhart, Antonín Přidal

**Affiliations:** 1 Univerzita Hradec Králové, Faculty of Science, Department of Biology, Rokitanského 62, Hradec Králové, Czech Republic Univerzita Hradec Králové, Faculty of Science, Department of Biology, Rokitanského 62 Hradec Králové Czech Republic; 2 Súťažná 10, Bratislava, Slovakia Súťažná 10 Bratislava Slovakia; 3 Global Change Research Institute of the Czech Academy of Sciences, Lipová 1789/9, , České Budějovice, Czech Republic Global Change Research Institute of the Czech Academy of Sciences, Lipová 1789/9 , České Budějovice Czech Republic; 4 Charles University in Prague, Faculty of Science, Department of Zoology, Viničná 7, Prague, Czech Republic Charles University in Prague, Faculty of Science, Department of Zoology, Viničná 7 Prague Czech Republic; 5 Koppert s.r.o., Komárňanská cesta 13, Nové Zámky, Slovakia Koppert s.r.o., Komárňanská cesta 13 Nové Zámky Slovakia; 6 Institute of Parasitology, Biological Centre CAS, Branišovská 31, České Budějovice, Czech Republic Institute of Parasitology, Biological Centre CAS, Branišovská 31 České Budějovice Czech Republic; 7 Mendel University in Brno, Faculty of AgriSciences, Department of Zoology, Fishery, Hydrobiology and Apidology, zemědělská 1, Brno, Czech Republic Mendel University in Brno, Faculty of AgriSciences, Department of Zoology, Fishery, Hydrobiology and Apidology, zemědělská 1 Brno Czech Republic

**Keywords:** Apoidea, Colletidae, ivy-bee, species occurrence, *Hedera helix*

## Abstract

*Colletes hederae* Schmidt & Westrich, 1993 is a cryptic bee species from the *C. succinctus* species-group. The previous occurrence and spreading of this species were predominantly in south-western Europe. To determine if the species was spreading in Slovak territory, *Hedera helix* was monitored from autumn 2015. The ivy-bee was first recorded in Slovakia during autumn 2017. This species is widespread inside and around Bratislava; however, it was not recorded under this study in any sites located eastwards. In the Czech Republic, it was not recorded in the south-east part of the country in 2017–2019. In 2020, the occurrence of this species was confirmed in many localities in the south of the country and strong populations were discovered, especially in the towns Znojmo and Mikulov. The populations likely originated from neighbouring Austria, where this species was discovered in 2006 and the localities are usually less than 100 km away from Czech and Slovak localities. A further survey could map a route of the northwards spread of this species.

## Introduction

*Colletes hederae* Schmidt & Westrich, 1993 is a cryptic species from the *C. succinctus* species-group with minute morphological differences useful for its identification (Amiet et al. 1999, Kuhlmann et al. 2007); thus, the species was described recently according to type material from north-western Croatia (Istria), north and central Italy, south-western Germany and south-eastern France (Schmidt and Westrich 1993). Currently, the rapid spread of this species has been recorded in Europe (Hopfenmüler 2014). The discovery of this plasterer bee species was, on the one hand, facilitated by its plant preference for ivy (*Hedera helix*) (Schmidt and Westrich 1993) and, on the other hand, impeded by confusing it with *C. succinctus* (Linnaeus, 1758) (Amiet et al. 1999) or *C. halophilus* Verhoeff, 1944 (Amiet et al. 1999, Kuhlmann et al. 2007, Zenz et al. 2021). The progress in the spread of the species after its discovery can be assumed from individual faunistic studies (Wiering 1999, Cross 2002, Ortiz-Sanchez 2009, Standfuss 2009, Roberts and Vereecken 2010, Teppner et al. 2009, Burger 2010, Voigt and Szalai-Dobosné 2019, Saure et al. 2019, Saure 2020), as it is obvious also from the distribution maps created by Kuhlmann et al. (2007) or Vereecken et al. (2009). The current species distribution is as follows: Austria, Belgium, Bosnia, Croatia, France, Germany, Greece, Italy, Montenegro, Netherlands, Serbia, Slovenia, Spain, UK and Channel Islands (all summarised by Kuhlmann et al. 2007), Switzerland (Amiet et al. 1999), Russia: Crimea (Proshchalykin et al. 2017) and Hungary (Voigt and Szalai-Dobosné 2019). Interestingly, the spread likely travels in two directions – the species is enlarging its area to the northwest (from southern to towards the UK in the north-west (Vereecken et al. 2006, Vereecken et al. 2009)) and to the east in the south Europe (from Italy and Croatia to Serbia, Greece, Bosnia and Crimea) (Jacobi et al. 2015, Zettel and Wiesbauer 2014).

The reasons for which the species enlarges its distribution area are not well understood. Several indications are pointing to the impact of the unusually high temperatures at the turn of summer and autumn resulting from global climatic change (Frommer 2010, Hopfenmüler 2014). Teppner et al. (2009) and Frommer (2010) observed that *C. hederae* creates only a local settlement “with larger distribution gaps” during its immigration and only a subsequent mass spread closes these gaps. Similar experiences were noted by Zettel and Wiesbauer (2014) in Lower Austria.

There are exceptions from the above mentioned northwesterly direction of the species as follows: the northern direction in Austria and Hungary (Teppner et al. 2009, Voigt and Szalai-Dobosné 2019) eastern directions recorded in Greece (Standfuss 2009) and on the Crimea Peninsula (Proshchalykin et al. 2017). With regard to these faunistic records and expected eastwards expansion (Vereecken et al. 2009), we observed bees on ivy (*H. helix*) in many localities situated in the southern part of Slovakia from the season in 2015 and in the south of Moravia (eastern part of the Czech Republic according to Bogusch et al. 2007) from the season in 2017. Additionally, we tried to discover this species in the south of Bohemia (western part of the Czech Republic according to Bogusch et al. (2007)) in 2020. This contribution aims to report the first recorded occurrence of *C. hederae* in Slovakia in the year 2017 and the Czech Republic in the year 2020.

## Material and methods

*Hedera helix* was observed within the blooming period (August to October) due to the expected spreading of *C. hederae* to Slovakia from autumn 2015. These observations were carried out in urban areas and always in places with a connection to gardens (hedge, overgrown fence, small garden building) or parks. Cemetery walls were also frequently studied localities. Altogether, we observed flower visitors on the ivy at 42 localities in Slovakia between the years 2015–2020 (Tables 1, 2).

In the Czech Republic, sites with flowering ivy were found and searched for the occurrence of *C. hederae*. All localities were situated in the south of the country, usually very close to the border with Austria and Slovakia. In 2017, we investigated localities in Kostice, Lanžhot and Břeclav and, in 2018–2019, in Nové Hrady randomly, but the species was not discovered. In 2020, the species was recorded in Kostice and Břeclav and many other localities in the Region of Moravia, as well as in two localities in the south of Bohemia. Altogether, we studied ivy flower visitors in 44 localities in the Czech Republic (16 in Bohemia and 28 in Moravia). In Tables 1, 2, all observed sites are listed with the coordinates, collector names and numbers of recorded specimens at each locality.

We observed the insects flying around and sitting on flowers of ivy during the warmest part of the day (between 11 a.m. and 4 p.m.) from the end of August until mid-October. Observations were only carried out at temperatures higher than 18°C in warm weather, with sunny or only partially cloudy skies, with no rain and only slight wind. Each locality was studied for 45 minutes or a shorter time if the bee were discovered earlier. Specimens of *C. hederae* were determined in the field and several individuals (usually one pair) from each locality were collected and placed into 75% alcohol for the collection and further revision. The identity of all collected specimens was checked in the laboratory by P. Bogusch, J. Lukáš, A. Přidal, M. Šlachta and J. Straka. The collected individuals from Slovakia are deposited in the collection of P. Bogusch, J. Lukáš, A. Přidal and J. Straka and individuals from the Czech Republic in the collections of P. Bogusch, A. Přidal and M. Šlachta. Maps were created using QGIS 3.6 software, localities and bees on ivy, were photographed using a mobile phone camera.

## Results

**Figure 1.**
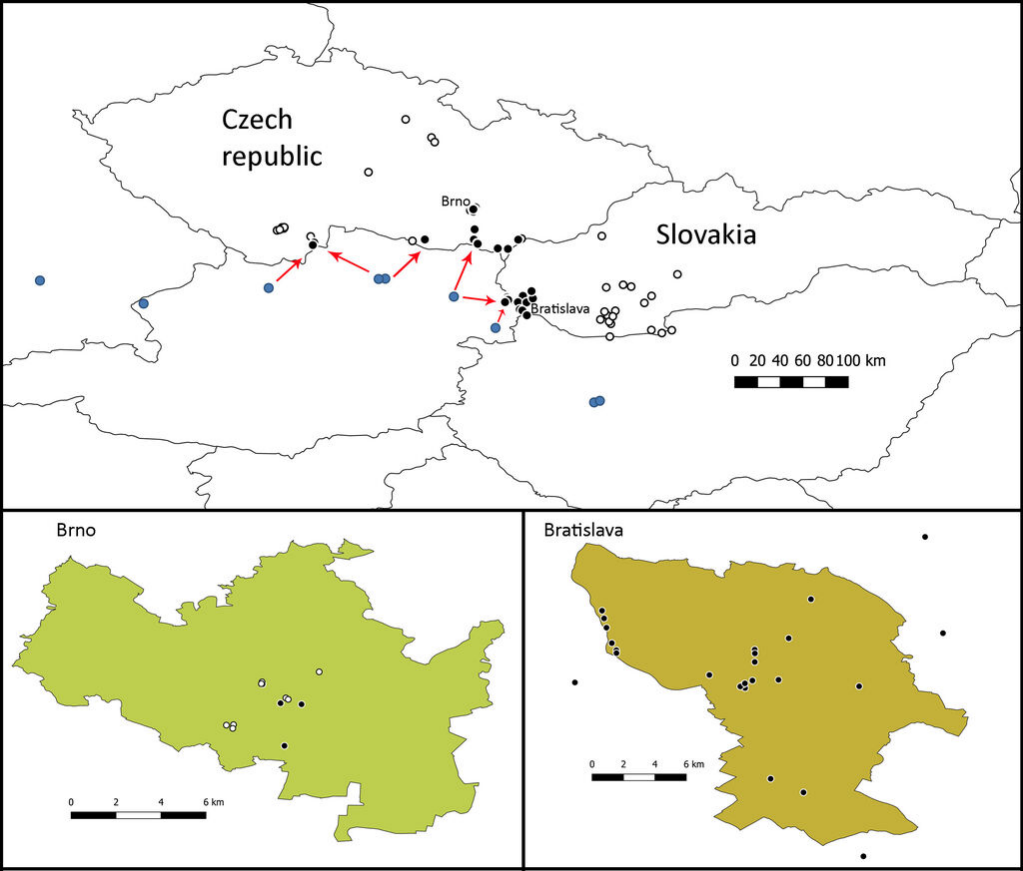
Map of the Czech Republic and Slovakia. Localities with the occurrence of *Colletes hederae* marked by a full circle and an empty circle were used for indication of a locality without record of the species. The nearest sites in Austria, Germany and Hungary are marked by blue circles and the possible routes of spreading towards Slovakia and the Czech Republic are shown by arrows. The species occurrence in the largest towns Brno (Czech Republic) and Bratislava (Slovakia) is shown in the maps under the top map.

We discovered adults of *C. hederae* in 19 localities in the Czech Republic (two in Bohemia and 17 in Moravia) and in 24 localities in Slovakia (Fig. 1, Table 1). The species was first discovered in Slovakia in 2017 in two localities: (1) Devínská Nová Ves, Sandberg and (2) Bratislava, Rača, Potočná Street. In the first locality, more than ten males and females flying around and on flowers of ivy were recorded, as well as males in the nearby locality of Sandberg Natural Reserve (J. Straka and P. Bogusch, observed and collected). In the next years, the species was discovered in an additional 22 localities, which were situated all around the Slovak capital Bratislava and the furthest locality from the first record was Pezinok (21 km north). In total, two records of *C. hederae* were documented also from Hungary and Austria and both are really close to the localities situated inside or around Bratislava. Interestingly, no specimens of *C. hederae* were observed towards the east and north directions in the localities around Trenčín, Nové Zámky, Komárno and Banská Štiavnica (Fig. 1, Table 2).

**Figure 2.**
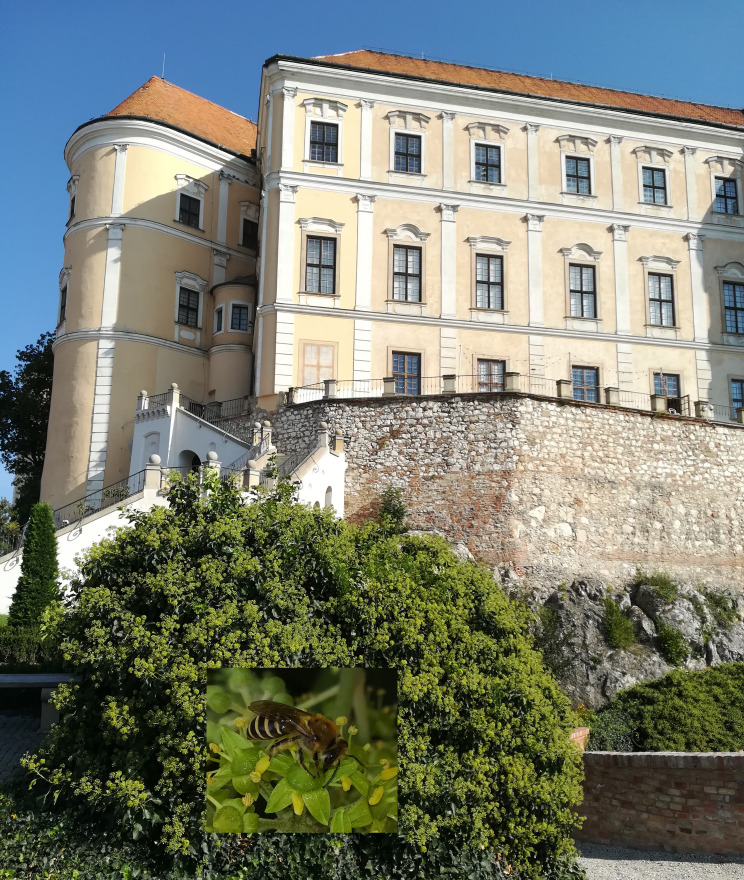
Locality of large population of *Colletes hederae* in Mikulov Castle (Czech Republic). Details of a female on flowers of ivy (*Hedera helix*). Photographed by P. Bogusch.

In the Czech Republic, the species was first discovered in 2020 in 17 localities in the south of Moravia and in two localities in the south of Bohemia. The first record was from Znojmo, where the species was observed on flowering ivy in the garden of the administration centre of Podyjí National Park. Here, a strong population was discovered and many females were collecting pollen. Similarly, large populations were recorded in two additional sites in the same town and in Mikulov around a distance of 40 km from Znojmo, where the species was recorded in all five checked sites, forming very large populations (Fig. 2). In all other sites, the species was found only in smaller numbers of individuals and probably is new there, in contrast to Mikulov and Znojmo, where the existence of large populations suggests that the bee has likely occurred there for a longer time. We searched for this species at several sites in 2017, 2018 and 2019, but *C. hederae* was not found. It was surprising to find *C. hederae* in four sites in Brno about 40 km north of Mikulov, but only several individuals were recorded and it is probable that the species has only occurred at these localities for a short amount of time. Recording this species in Brno required patience, as catching two individuals involved a 45-minute wait. These localities in Brno are the northernmost sites of occurrence of *C. hederae* in the Czech Republic.

In Bohemia, *C. hederae* was recorded in two localities in the southernmost part of the country, in relatively small populations. These localities are situated in the small town, Nové Hrady and the village, Byňov. The species was not recorded in the city České Budějovice and nearby areas, although seven sites were checked there for its occurrence.

## Discussion

The discovery of *C. hederae* in the Czech Republic and Slovakia is the latest proof of the spread of this species in northern and eastern directions. Since the species was not recorded in the sites of occurrence in Slovakia in 2015 and the Czech Republic in 2017–2018, we can suppose that *C. hederae* is a very new representative of the fauna of both countries. Czech and Slovak populations most likely originate from Austria, where *C. hederae* was first recorded in Vienna in 2006 (Teppner et al. 2009, Gusenleitner et al. 2012). It is quite surprising that the species was only discovered in the south of Slovakia (Bratislava is only about 50 km from Vienna) after such a long time – 11 years later and in Mikulov (80 km from Vienna) 14 years later. Perhaps, the weather conditions for spreading (Frommer 2010) were more suitable in the last few seasons, which were exceptionally warm in both winter and summer in Central Europe (Zahradníček et al. 2021). Spreading to Znojmo could be more difficult, whilst the species was recorded nearby in Austria, around Linz, but much later than in Vienna (Ebmer et al. 2018). While dispersal to Mikulov and Bratislava would be through warm lowland regions, the expansion to Znojmo had to partially pass through hilly and afforested areas. Similarly, Bohemian populations likely also came from these locations.

Although *C. hederae* occurs in other neighbouring countries, it is evident that Czech and Slovak populations originated from Austria. In Germany, the species was first discovered in 1991 (Schmidt and Westrich 1993), but following this, it was spreading especially in the south-western parts of the country and migration was more towards the north than the east (Jacobi et al. 2015). In recent studies, the nearest locality to the Czech Republic is Freising, where the species was recorded in 2015 (Fleischmann 2019). It is situated around 150 km from the German/Czech border and 210 km from the nearest localities of *C. hederae* in the Czech Republic. Newer German records (2017–2019) are from Berlin and Halle in central to northern Germany (Saure et al. 2019, Saure 2020), but these localities are far from the Czech localities of *C. hederae*. Additionally, in Hungary, the species was first recorded in 2016 near Balaton Lake (Voigt and Szalai-Dobosné 2019), which is even further from Slovak localities than the localities in Austria (140 km from Bratislava).

The sudden occurrence of *C. hederae* in the Czech Republic and Slovakia is somewhat similar to the situation in Graz, Austria (Teppner et al. 2009). With respect to the monitoring of many sites in the south of both countries with negative results, the species spreading seems to be not so “stormy” as Gusenleitner et al. (2012) recorded in Austria (“… seitdem in stürmischer Ausbreitung”). Czech and Slovak populations are usually small, which can relate to their relatively recent origin (most of the localities have been inhabited by ivy bee probably for one or two years). Strong populations in Mikulov and Znojmo can be explained by an earlier colonisation of both towns. Both towns probably provide suitable habitats for this bee – they are situated in the warmest parts of the country, both on south-orientated hillsides, with many possible vertical or semi-vertical clayish or loess walls for nesting and with medieval entrenchment and historical buildings, richly overgrown with *Hedera helix.* Bratislava has quite similar characteristics and the populations of *C. hederae* are large there, too.

**Figure 3.**
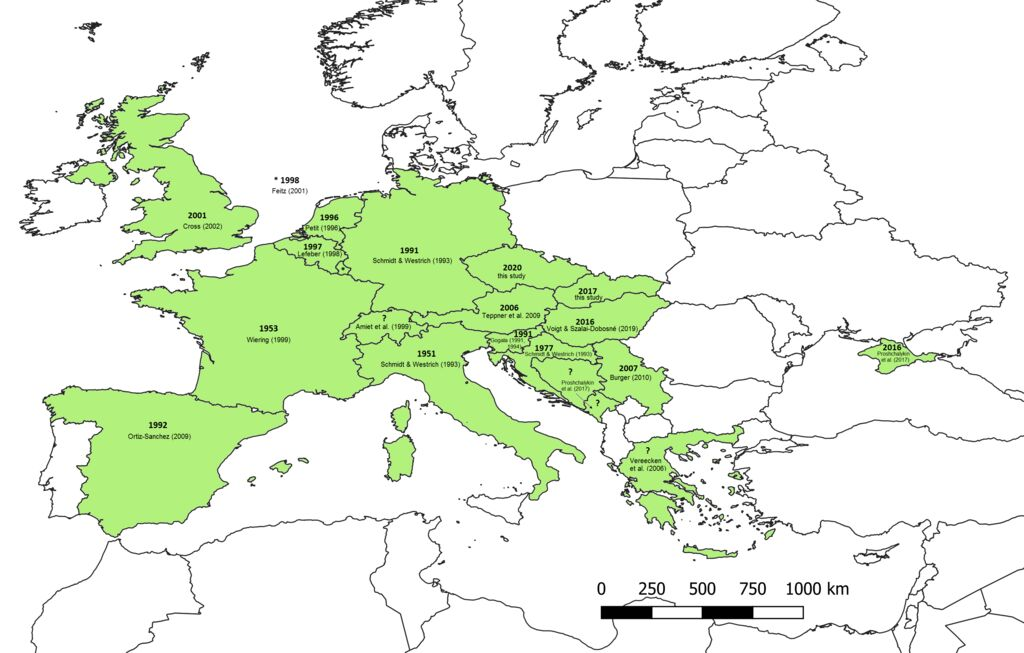
Distribution range map of *Colletes hederae* (green background) with the year of the first record/specimen (in the case of publishing) and references for each country (Feitz 2001, Gogala 1991, Gogala 1994, Lefeber 1998, Petit 1996).

Interestingly, the species occurs in Greece, where it is numerous on Lesbos Island, as well as occurring in the mainland (Vereecken et al. 2006, Vereecken et al. 2009) and Crimea in Russia (Proshchalykin et al. 2017). However, no records are known from Romania, although the monitoring of possible localities has been performed by specialists (B. Tomozei, pers. commun.) and a similar situation is present in Bulgaria (T. Ljubomirov, pers. commun.). The terrestrial routes of the spreading of the ivy-bee towards Crimea and Greece remain unclear and the spreading is certainly faster in some directions than in others. However, fragmented distribution ranges are not uncommon in the Mediterranean Basin and SE Europe (Kuhlmann, pers. commun.), as is the case for other species. Therefore, it might be that isolated populations exist in a wider area leaving large regions uninhabited. Kuhlmann et al. (2007) hypothesise that the ivy bee is under an unfinished dispersal process using arguments on distribution of the host plant, the recent colonisation of southern England and with postglacial dispersal limits of its cuckoo bee. In Fig. 3, the current distribution of the ivy-bee is depicted with a year of the first record for each country.

Although several authors declared that this species is observable from September to December (Bischoff et al. 2005, Roberts and Vereecken 2010, but in maritime climates in England), in more continental Central Europe, the species reacts to the colder weather in late autumn and winter and occurs at the latest till the end of October and only in the case of exceptionally warm and sunny weather. The flight activity on ivy was observed in Bratislava (streets Žižkova and Kráľovské údolie) on 23.9.2019 even after 6 p.m. Most of our observations are consistent with the species phenology, described by Bischoff et al. (2005) despite there being potential for the species activity until the end of October (Kuhlmann et al. 2007). The ivy-bee was recorded only on flowers of *H. helix*, but was likely due to the fact that it was not searched for on flowers of other plants. Males were also observed on flowers of other undetermined plants in Sandberg Nature Reserve and these probably do not specialise on feeding only on ivy nectar. Although Bischoff et al. (2005) reported that only pollen of *H. helix* was recorded in pollen loads of specimens observed in the UK, Müller and Kuhlmann (2008) and Westrich (2008) described *C. hederae* to be polylectic with a strong preference for *H. helix* (or mesolectic, according to Dötterl and Vereecken 2010). Identification of pollen loads from multiple females could clarify if the species also uses other plant species (e.g. Asteraceae, Ericaceae and Orobanchaceae) as a source of pollen (Müller and Kuhlmann 2008, Westrich 2011, Teppner and Brosch 2015), especially those flowering before the season of the ivy (Westrich 2008).

Further monitoring in the Czech Republic and Slovakia is desirable to gain a better understanding of the strategy in the spreading of this autumnal bee species, especially in the context of the possible dispersal towards the north and the east. While this species reached Slovakia in 2017 and the Czech Republic in 2020, it will be interesting to see if, when and in which direction this species will move to Poland. The Moravian Gate (Moravská brána) is considered as a migrating corridor to the north, not only for insect species (Sierka et al. 2008). However, Banaszak et al. (2017) analysed bee populations in “Góra Gipsowa” and indicated that the Moravian Gate does not play any part in the migration of southern bee species to Poland at present. Therefore, monitoring of *Colletes hederae* spreading through Moravia can help to explain the role of the Moravian Gate in spreading of insects to Poland in Central Europe.

## Conclusions

The spread of *C. hederae* evidently continues as confirmed by the new records from Slovakia (2017) and Czechia (2020). These and recent records from Austria and Hungary correspond with the north spread direction. It is not clear why, until recently, the ivy bee spread predominantly northwesterly. We hypothesise that spreading to the north could be supported by the several last warm seasons (namely autumns) with a higher temperature than their usual average. Monitoring of the continuation of the spread of the ivy bee is important, not only to recognise its ecological limits, but also to verify the zoogeographic importance of the Moravian Gate as a migration route.

## Figures and Tables

**Table 1.** Records of *Colletes hederae.* (N/E = northern latitude/eastern longitude; Lgt. = recorded by; M/F = male/female)

**Country**	**Town (Locality)**	**Coordinates (N/E)**	**Lgt.**	**Date**	**Sex (M/F)**
CZ	Byňov (way to Byňovský pond)	48.8187°/14.7998°	M. Šlachta	23–24.09.2020	5/0
CZ	Nové Hrady (Vitorazská)	48.7902°/14.7837°	M. Šlachta	1–4.10.2020	0/4
CZ	Brno (central cemetery)	49.1680°/16.5939°	A. Přidal	22.09.2020	1/2
CZ	Brno (Kraví Hora)	49.2056°/16.5805°	A. Přidal	22.09.2020	2/0
CZ	Brno (Špilberk)	49.1933°/16.6042°	A. Přidal	23.09.2020	0/2
CZ	Brno (Žlutý kopec)	49.1940°/16.5915°	A. Přidal	23.09.2020	1/0
CZ	Břeclav (cemetery wall)	48.7526°/16.8669°	P. Bogusch	15.09.2020	3/0
CZ	Dolní Dunajovice (cemetery wall)	48.8523°/16.5982°	P. Bogusch	14.09.2020	3/2
CZ	Hodonín (Velkomoravská)	48.8516°/17.0975°	P. Bogusch	15.09.2020	2/0
CZ	Kostice (Náměstí osvobození)	48.7469°/16.9815°	P. Bogusch	15.09.2020	3/0
CZ	Mikulov (around cemeteries)	48.8104°/16.6381°	P. Bogusch	15.09.2020	>10/>10
CZ	Mikulov (Castle)	48.8065°/16.6365°	P. Bogusch	15.09.2020	>10/>10
CZ	Mikulov (Dietrichstein chamber)	48.8064°/16.6399°	P. Bogusch	15.09.2020	>10/>10
CZ	Mikulov (Kamenný řádek)	48.8072°/16.6416°	P. Bogusch	15.09.2020	>10/>10
CZ	Mikulov (Novokopečná)	48.8043°/16.6409°	P. Bogusch	15.09.2020	>10/>10
CZ	Vranovice (near the square)	48.9678°/16.6062°	P. Bogusch	15.09.2020	2/0
CZ	Znojmo (administration center)	48.8586°/16.0444°	P. Bogusch	14.09.2020	>10/>10
CZ	Znojmo (Dyjská)	48.8510°/16.0496°	P. Bogusch	14.09.2020	3/8
CZ	Znojmo (under the terraces)	48.8532°/16.0455°	P. Bogusch	14.09.2020	8/>10
SK	Bratislava (Deví/Hadia cesta)	48.1636°/17.1023°	J. Lukáš	05.09.2019	0/1
SK	Bratislava (Deví/Hradná)	48.1929°/16.9759°	J. Lukáš	21.09.2018	0/1
SK	Bratislava (Deví/Slovanské nábrežie)	48.1737°/16.9843°	J. Lukáš	21.09.2018	0/1
SK	Bratislava (Karlova Ves, Liščie údolie)	48.1525°/17.0636°	J. Lukáš	20.09.2018	1/12
SK	Bratislava (Kráľovske údolie)	48.1414°/17.0941°	J. Lukáš	21.09.2018	>10/1
SK	Bratislava (Nové Město, Brečtanova)	48.1742°/17.1023°	J. Lukáš	26.09.2019	0/3
SK	Bratislava (Nové Město, Račianska)	48.1709°/17.1024°	J. Lukáš	19.09.2018	1/1
SK	Bratislava (Nové Město, way to Kamzík)	48.1838°/17.1313°	J. Lukáš	18.09.2018	0/1
SK	Bratislava (Ondrejský cemetery)	48.1484°/17.1226°	J. Lukáš	16.09.2020	0/1
SK	Bratislava (Palisády)	48.1478°/17.1003°	M. Wiezik	18.09.2020	0/2
SK	Bratislava (Rača, Potočná)	48.2172°/17.1503°	J. Lukáš	29.09.2017	0/1
SK	Bratislava (Staré Město, Fialkove údolie)	48.1446°/17.0930°	J. Lukáš	21.09.2018	1/1
SK	Bratislava (Staré Město, Mikulášsky cemetery)	48.1427°/17.0900°	J. Lukáš	18.09.2019	1/2
SK	Bratislava (Staré Město, Mudroňova)	48.1453°/17.0939°	J. Lukáš	27.09.2018	0/1
SK	Bratislava (Vrakuňa cemetery)	48.1428°/17.1914°	J. Lukáš	16.09.2020	0/2
SK	Devínská Nová Ves (6. Apríla square)	48.2074°/16.9721°	J. Lukáš	21.09.2018	1/1
SK	Devínská Nová Ves (near Sandberg)	48.2008°/16.9739°	J. Straka	31.08.2017	4/0
SK	Devínská Nová Ves (near Sandberg)	48.2008°/16.9739°	P. Bogusch	05.09.2017	6/3
SK	Devínská Nová Ves (Waitov quarry)	48.1797°/16.9804°	J. Lukáš	21.09.2018	0/1
SK	Ivanka při Dunaji (Potocká)	48.1882°/17.2628°	J. Lukáš	16.09.2020	0/2
SK	Jarovce (cemetery wall)	48.0639°/17.1159°	J. Lukáš	15.09.2020	2/1
SK	Limbach (Športová)	48.1712°/16.9844°	J. Lukáš	29.09.2019	0/2
SK	Pezinok (Grinava, Štúrova)	48.2705°/17.2476°	J. Lukáš	29.09.2019	0/2
SK	Rusovce (cemetery wall)	48.0522°/17.1439°	J. Lukáš	15.09.2020	4/1
A	Hainburg (cemetery wall)	48.1461°/16.9490°	J. Lukáš	30.09.2018	0/1
H	Rajka (cemetery wall)	47.9976°/17.1950°	J. Lukáš	23.09.2019	0/1

**Table 2.** Locations without recorded occurrence of *Colletes hederae*. (N/E = northern latitude/eastern longitude; Obs. = observed by)

**Country**	**Town**	**Locality**	**Coordinates (N/E)**	**Observed**	**Date**
CZ	České Budějovice	E. Rošického Street	48.9893°/14.4362°	J. Erhart	23.09.2020
CZ	České Budějovice	Lipová Street	48.9778°/14.4559°	J. Erhart	23.09.2020
CZ	České Budějovice	near football ground	48.9823°/14.4475°	J. Erhart	23.09.2020
CZ	České Budějovice	Plzeňská	48.9895°/14.4631°	J. Erhart	23.09.2020
CZ	České Budějovice	V Oblouku	48.9908°/14.4614°	J. Erhart	23.09.2020
CZ	Hradec Králové	terraces	50.2087°/15.8293°	P. Bogusch	22.09.2020
CZ	Jiterní Ves	at crossroad	48.8881°/14.7590°	M. Šlachta	23.09.2020
CZ	Kaliště u Lípí	centre	48.9563°/14.3808°	J. Erhart	23.09.2020
CZ	Lipnice nad Sázavou	near the square	49.6132°/15.4106°	P. Bogusch	03.10.2020
CZ	Mokré	margin	48.9649°/14.4108°	J. Erhart	23.09.2020
CZ	Nové Hrady	at pond Sedlákovec	48.7907°/14.7836°	M. Šlachta	23.09.2020
CZ	Nové Hrady	Vallon Cheri apartments	48.7900°/14.7757°	M. Šlachta	23.09.2020
CZ	Rzy		50.0040°/16.1205°	P. Bogusch	27.09.2020
CZ	Vysoké Mýto	main road	49.9522°/16.1563°	P. Bogusch	22.09.2020
CZ	Brno	Černá Pole, Botanical Garden	49.2131°/16.6150°	A. Přidal	23.09.2020
CZ	Brno	Nový Lískovec	49.1809°/16.5629°	A. Přidal	24.08.–27.09.2020
CZ	Brno	Nový Lískovec	49.1786°/16.5624°	A. Přidal	24.08.–27.09.2020
CZ	Brno	Nový Lískovec	49.1806°/16.5585°	A. Přidal	24.08.–27.09.2020
CZ	Brno	Stránice	49.2070°/16.5802°	A. Přidal	22.09.2020
CZ	Brno	Stránice	49.2059°/16.5798°	A. Přidal	22.09.2020
CZ	Brno	Špilberk	49.1972°/16.5947°	A. Přidal	23.09.2020
CZ	Brno	Špilberk	49.1963°/16.5962°	A. Přidal	23.09.2020
CZ	Hodonín	Na Výhoně	48.8628°/17.1352°	P. Bogusch	15.09.2020
CZ	Lukov	Nový Hrádek	48.8372°/15.9059°	P. Bogusch	15.09.2020
CZ	Znojmo	Granické Valley	48.8553°/16.0406°	P. Bogusch	14.09.2020
SK	Andovce	Novozámecká	47.9950°/18.1042°	P. Šima	2017–2020
SK	Bánov		48.0487°/18.1915°	P. Šima	03.10.2020
SK	Banská Štiavnica	Starozámocká	48.4601°/18.8915°	P. Šima	2017–2020
SK	Belá		47.8319°/18.5973°	P. Šima	2017–2020
SK	Beladice	Pustý Chotár	48.3417°/18.2796°	P. Šima	10.10.2020
SK	Chľaba		47.8295°/18.8273°	P. Šima	2017–2020
SK	Imeľ		47.9020°/18.1413°	P. Šima	03.10.2020
SK	Komárno		47.7574°/18.1298°	P. Šima	2017–2020
SK	Komoča		47.9500°/18.0232°	P. Šima	03.10.2020
SK	Levice		48.2163°/18.6000°	P. Šima	2017–2020
SK	Nesvady		47.9267°/18.1217°	P. Šima	03.10.2020
SK	Nitra		48.3129°/18.0894°	P. Šima	2017–2020
SK	Nové Zámky		47.9861°/18.1631°	P. Šima	2017–2020
SK	Ondrejovce		48.1376°/18.5187°	P. Šima	2017–2020
SK	Palárikovo		48.0392°/18.0698°	P. Šima	2017–2020
SK	Štúrovo		47.7978°/18.7158°	P. Šima	2017–2020
SK	Trenčín		48.8923°/18.0393°	J. Lukáš	2017–2020
SK	Vieska nad Žitavou	Mlyňany	48.3217°/18.3700°	P. Šima	10.10.2020
